# Impact of Exercise and Education in Adults of Lubbock, Texas: Implications for Better Lifestyle

**DOI:** 10.3389/fnagi.2016.00085

**Published:** 2016-05-10

**Authors:** Annette N. Boles, Hafiz Khan, Taylor A. Lenzmeier, Veronica A. Molinar-Lopez, James C. Ament, Kate L. TeBrink, Kathleen Stonum, Ruben M. Gonzales, P. Hemachandra Reddy

**Affiliations:** ^1^Garrison Institute on Aging, Southwest Campus, Texas Tech University Health Sciences CenterLubbock, TX, USA; ^2^Garrison Institute on Aging, Texas Tech University Health Sciences CenterLubbock, TX, USA; ^3^Department of Public Health, Texas Tech University Health Sciences CenterLubbock, TX, USA; ^4^Cell Biology and Biochemistry, Neuroscience/Pharmacology and Neurology Departments, Texas Tech University Health Sciences CenterLubbock, TX, USA

**Keywords:** aging, behavioral research, nutrition and exercise education, community based participatory research, intervention studies

## Abstract

The objective of our study was to evaluate the exercise and educational intervention in the city of Lubbock via GET FiT Lubbock (GFL) program. The GFL program was designed to increase exercise and educational opportunities, which positively impact health risk factors in Lubbock residents. The GFL program design included the recruitment of subjects to participate on a team that consisted of four individuals, each subject tracked their exercise minutes, and their educational session attendance. The tracking of exercise and educational sessions was done on the GFL website. Biometric testing was conducted pre- and post- intervention. The program was located within the Lubbock community in places that were close to their place of residence. The intervention included walking and educational sessions, including goal setting lectures, nutrition information, and exercise demonstrations. Study participants, included male and female adults who tracked their exercise time and educational sessions. Exercise minutes and educational session attendance were self-reported. Our data analysis revealed that significant difference was found between pre- and post- intervention measures, including weight, body mass index (BMI), high-density lipoprotein (HDL). Significant difference was found for weight, BMI, and HDL in females. Based on these findings, we conclude that the intervention showed positive effects on exercise and lifestyle.

## Introduction

The United States faces an epidemic of unhealthy behaviors. Modifiable risk factors such as obesity, high blood glucose, hypertension, and physical inactivity leads to chronic diseases (Centers for Disease Control and Prevention. National diabetes fact sheet, 2011; Centers for Disease Control and Prevention Chronic Disease Prevention and Health Promotion, 2015). Currently, more than one-third of adults in the U.S. are obese (Ogden et al., 2006). Nationally, 25.4% reported no leisure-time physical activity. While in Texas, the statistics reported are even higher at 27.2% (Centers for Disease Control and Prevention, State Indicator Report on Physical Activity, 2014). As a nation, the U.S. has gone from having no state with an obesity rate higher than 15% in 1990, to having no state with an obesity level lower than 21% today (Ogden et al., 2006; The State of Obesity^1^; CDC and U.S. Department Of Health and Human Services, Preventing Chronic Diseases, 2015). As a consequence, because the highest risk factor for developing type-2 diabetes is obesity, the incidence of diabetes has skyrocketed to over 25 million nationwide (Centers for Disease Control and Prevention. National diabetes fact sheet, 2011), with over 1.8 million in Texas (Prevalence of Diabetes in Texas and Bexar County, 2012). In Lubbock, the prevalence of diagnosed diabetes is ~33,000 residents and pre-diabetes is 85,000 residents (American Diabetes Association^2^). In addition, over 40% of Lubbock residents are diagnosed with hypertension (Carr, 2014), and 26% of the population is obese (Centers for Disease Control and Prevention Division of Nutrition, Physical Activity and Obesity^3^).

Organizations across the country have been implementing strategies to help combat these unhealthy trends. Strategies range from environmental changes to policy interventions. Environmental changes include increasing opportunities for individuals to use public facilities for increased opportunities. These include building walking trails at local parks and adding bike lanes and routes to protect individuals that would use active modes of transportation. One of the largest policy interventions is the addition of wellness programs to workplaces. These programs not only increase the health and morale of employees, they can also lower costs for the business (Stokes et al., 2006; Davis et al., 2009; Baicker et al., 2010). While these strategies provide good results, it stands to reason that communities across the country need to find creative ways to combat these unhealthy trends as a whole. Program strategies need to persuade the community to become more active and are simplistic enough to replicate in any setting. An organization that has made environmental policy change a priority and establishes programs that are easy to replicate in other settings, especially in disparate communities, is the Texas Tech University Health Sciences Center Garrison Institute on Aging (TTUHSC GIA). TTUHSC GIA created a program designed to use accountability, teamwork, and competition to help the community of Lubbock become a healthier place to live.

The purpose of our study was to evaluate the exercise and educational intervention in the city of Lubbock via GET FiT Lubbock (GFL) program. The GFL program was designed to increase exercise and educational opportunities, which positively impact health risk factors in Lubbock residents.

The GFL is an 8-week community-based competition in which teams of 4 earn points for exercise, weight loss, and attendance at community events can be implemented in both community and workplace settings. Participants register online or via paper registration form. Teams choose to compete in one of three categories based on the amount of time each team member commits to exercise every week. Categories include: Raider Rookie, each team member commits to exercising 150 min per week; Raider Power, each team member commits to exercising 270 min per week; and Raider Warrior, each team member commits to exercising 360 min per week.

These categories were based on the recommendations made by the American College of Sports Medicine (ACSM)^4^ for physical activity. The subjects in this study participated in the GFL program and used the tracking website. Participation in the research component was entirely optional and did not affect any part of the competition. Teams, as a whole, did not need to partake in the research study.

## Materials and methods

Lubbock County consists of more than 290,000 people. Among them the majority is Hispanic or Latino, which comprises 32.1, and 58.4% of residents are between the ages of 18 and 64 years. The average income (between 2010 and 2014) was $45,529 per year, and 17.7% of the population fell below the poverty line (U.S. Census Bureau^5^). Approximately, 33,000 people are diagnosed with diabetes each year, and 85,000 are diagnosed with pre-diabetes and 41.5% of the City of Lubbock population is diagnosed with hypertension (Carr, 2011). In addition, 26% of the population is obese (U.S. Census Bureau; Carr, 2014). Based on these statistics, it is apparent that active living opportunities and healthy eating education is needed.

### IRB approval

This study protocol was approved by TTUHSC IRB (Texas Tech University Health Sciences Center, Institutional Review Board) NUMBER: L15-158; IRB APPROVAL DATE: 07/06/2015.

### Program study description

**Figure 1:**
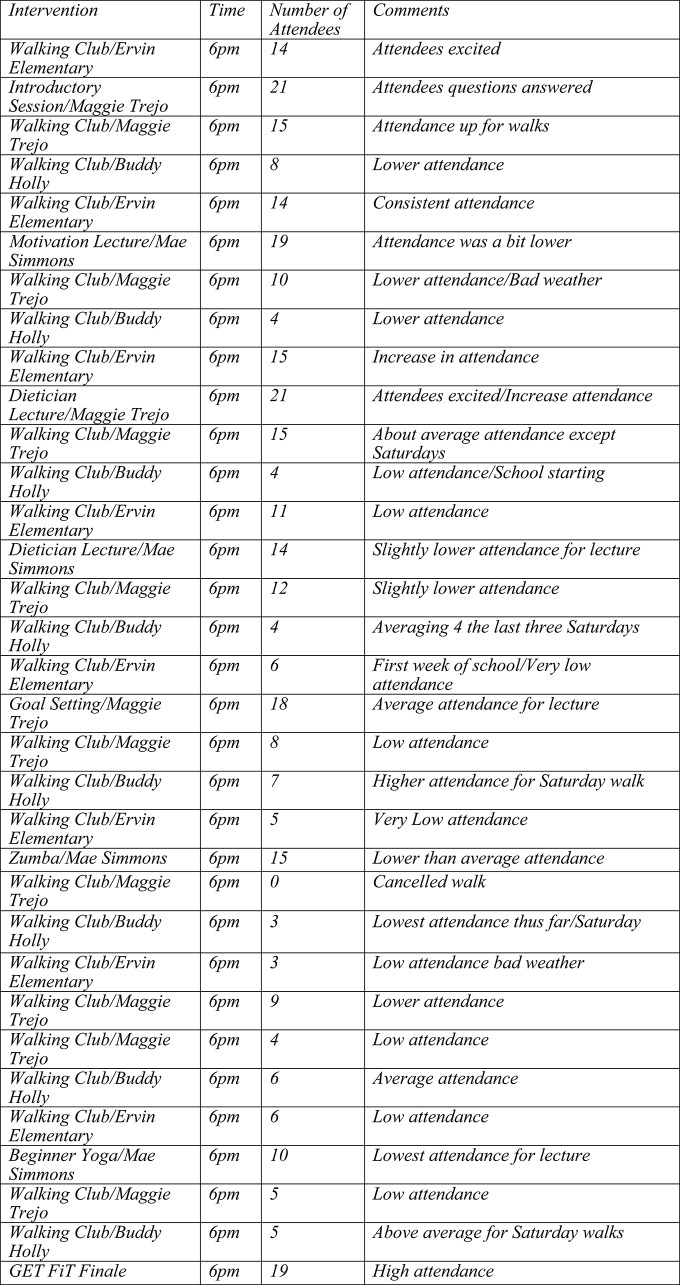
**Summary of GFL study participants' physical activity**.

The GFL program was the intervention used in the study. The intervention includes an exercise and educational component. (See Figure 1 for information about the physical activity component.) Subjects involved in GFL create a team of four people, elect a team captain, complete the necessary paperwork, and use the website (http://www.healthylubbock.org/getfit) to track exercise time, and attendance to educational sessions. Subjects earn 1 point per minute of exercise, 50 points per each percent of weight loss, and 50 points for attending educational sessions. The teams with the most number of points at the end of the 8 weeks won the challenge.

The study aimed to decrease modifiable risk factors of subjects that participated in GFL. The overall goal was to determine how the GFL program impacted subjects' cholesterol levels, glucose levels, blood pressure levels, weight, BMI, and body fat percentage. Exercise time was self-reported, and it was done independently and/or in the walking club. Educational session attendance was also self-reported. However, sign in sheets were at each educational session. Therefore, study personnel were able to verify attendance at education sessions.

### Recruitment

After final IRB approval of the study, study personnel began recruitment by posting approved fliers in various locations in the community. The primary areas of focus included community centers, senior centers, grocery stores, local businesses, and clinics. Informational sessions were also held at community centers and senior centers. Emails with flier attachments and information about the program were distributed to previous GFL participants.

### Registration

The community members who were interested in the program, registered online at www.healthylubbock.org/getfit or via paper registration form. If participants chose to be involved in the research study, the study personnel were alerted of the participant's involvement via emails, mails, or telephones. The study personnel then called each participant to confirm their participation and set an appointment to conduct biometric screenings, completion of a consent form, a behavioral survey, and the inclusion and exclusion criterion information sheet. The questionnaire is included in the Appendix.

There were three designated locations to complete the necessary paper works and have the biometric testing conducted. Potential subjects were also notified that they would receive a $7.00 gift card after their initial screenings and $7.00 gift card after their final screening.

### Procedures of initial biometric screenings

The initial biometric screenings were conducted at the following sites: Maggie Trejo Super Center (MTSC), Copper Rawlings Center (CRC), and the GIA. Study participants had the following tests conducted: lipid panel (testing subject's cholesterol) and A1C (testing subject's blood glucose). These tests required a 12-h fasting period (No food or drink. Water was allowed, and no vigorous exercise for 12 h prior) before the blood draw. Other tests included: blood pressure, body mass index (BMI) and body fat percentage. The first (baseline) screening conducted by the Texas Tech University Health Sciences Center (TTUHSC) Clinical Research Institute (CRI) occurred at the beginning of the study. The CRI staff performed the lipid panel and A1C testing. The University Medical Center (UMC) Lab Department analyzed the blood draws. The TTUHSC Garrison Institute on Aging study personnel conducted the blood pressure, body fat percentage and BMI (height and weight) tests. The study participants were not found to have any abnormal vital signs or laboratory results during this study according to the UMC Lab Department. The distribution of age of the participants is given in Table 1 in the Results Section.

**Table 1:** **Age distribution of participants**.

**Age groups**	**Frequency**	**Percent**	**Cumulative percent**
21–30	9	17.0	17.0
31–40	13	24.5	41.5
41–50	10	18.9	60.4
51–60	9	17.0	77.4
61–70	10	18.9	96.2
71–80	1	1.9	98.1
81+	1	1.9	100.0
Total	53	100.0	

### Procedures of post-testing biometric screenings

The post-testing of biometric screenings was conducted during the end of the 8th week of the study. The post-testing screenings were similar to the initial screenings; testing was not conducted at the MTS and a behavioral survey was not conducted. Testing was conducted at CRCC and GIA. Study participants had the following tests conducted: lipid panel (testing subject's cholesterol) and A1C (testing subject's blood glucose). These tests required a 12-h fasting period (no food or drink, water was allowed, and no vigorous exercise for 12 h prior) before the blood draw. Other tests included: blood pressure, body mass index (BMI) and body fat percentage. Similar to the pre-testing, TTUHSC CRI staff performed the lipid panel, blood pressure, and A1C testing. The UMC Lab Department analyzed the blood draws. The same study personnel from TTUHSC GIA conducted the body fat percentage, blood pressure, and BMI [Body Mass Index, weight kg/(height m)^2^] as during the initial testing.

### Sample selection

Our target population was minorities within the Lubbock, West Texas population, which included primarily the African American and Hispanic ethnicity groups within our community. The sample size was calculated by using G^x^Power software version 3.1.1. It was determined that 28 participants would be sufficient to compare pre and post measurements with α = 0.05, effect size = 0.50, and power = 80% when running the paired samples *t*-test. IBM SPSS Statistics version 23 was used for the data analysis. This software was used to obtain descriptive and inferential statistics of the variables for the pre and post measurements.

## Results

**Figure 2:**
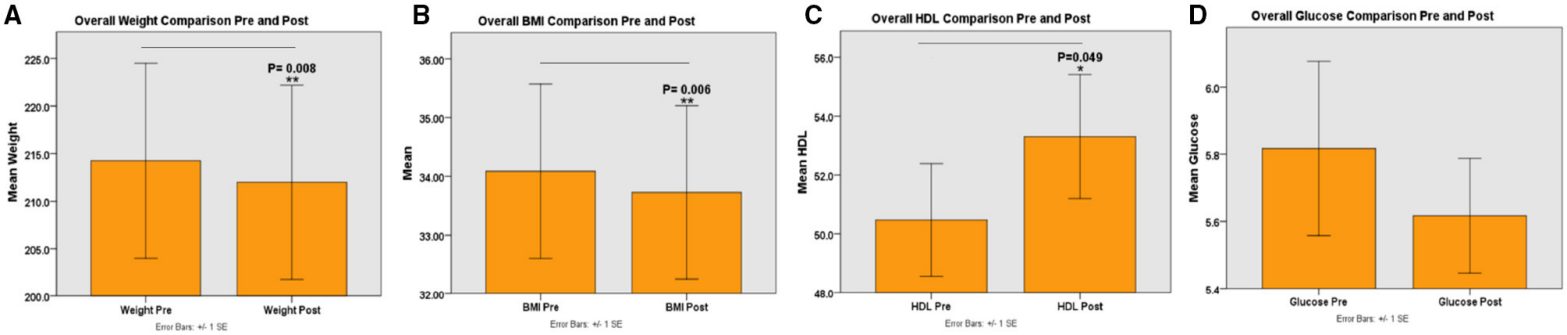
**Shows Pre- and Post-interventions measures, including weight, body mass index, high-density lipoprotein. (A)** Represents overall body weight comparisons between pre and post-interventions; **(B)** Represents overall body mass index between pre and post-interventions; **(C)** Represents overall high density lipoprotein between pre and post-interventions; and **(D)** Represents overall glucose comparison between pre and post-interventions. ^*^P <0.05, ^**^P <0.01.

Our data analysis revealed that significant difference was found between pre- and post- intervention measures, including weight, body mass index (BMI), high-density lipoprotein (HDL) (Figure 2). Significant difference was found for weight, BMI, and HDL in females but not in males. Details are given below.

### Pre-measurement

For the weight variable (*n* = 52) descriptive statistics included a range (R) = 112–369.5 pounds, average = 209.22, and standard deviation (SD) = 63.42 pounds. The quartiles for weight were obtained as Q1 = 161, Q2 = 199.5, and Q3 = 244.5, respectively. For the BMI variable (52) R = 20.19–57.83, average = 33.29, and SD = 9.17. The quartiles for BMI were obtained as Q1 = 26.07, Q2 = 31.67, and Q3 = 37.36.

There was a decrease in participants due to poor veins in the measurement of LDL (*n* = 47), HDL (*n* = 47), and Glucose (*n* = 46). The LDL variable included *R* = 67–185, average = 119.15, and SD = 28.35. The quartiles for LDL were obtained as Q1 = 98, Q2 = 119, and Q3 = 141. The HDL variable had R = 27–72, average = 50.17, and SD = 12.29. The quartiles for HDL were obtained as Q1 = 40, Q2 = 51, and Q3 = 61. The cholesterol variable had R = 108–246, average = 178.7, and SD = 33.28. The quartiles for cholesterol were obtained as Q1 = 162, Q2 = 175, and Q3 = 201. The glucose variable had R = 4.6–13.1, average = 5.69, and SD = 1.36. The quartiles for glucose were obtained as Q1 = 5.18, Q2 = 5.4, and Q3 = 5.7.

The participants increased in the measurement of body fat, systolic blood pressure, and diastolic blood pressure (*n* = 52) since these were obtained from all participants at the screenings. The body fat variable was R = 21.5–57.5, average = 39.15, and SD = 10.13. The quartiles for percent body fat were obtained as Q1 = 30.83, Q2 = 40.7, and Q3 = 47.15. Systolic blood pressure had R = 97–174, average = 126.54, and SD = 16.45. The quartiles for systolic blood pressure were obtained as Q1 = 113, Q2 = 132, and Q3 = 136. For the diastolic blood pressure variable R = 63–104, average = 81.31, and SD = 10.01. The quartiles for diastolic blood pressure were obtained as Q1 = 73.25, Q2 = 82, and Q3 = 88.5. It is obvious from Table 1, the most frequent age group is 31–40, and the lowest is 71^+^. The age group 21–70 comprises 96.2% of the study population. Table 2 describes the distribution of gender, race, and ethnicity. Sixty-nine percent were female and thirty-one percent were male of the participants. The Caucasian/White group had the highest ethnicity group and African American/Black contributed the lowest.

**Table 2:** **Summary of demographics of study participants**.

	**Frequency**	**Percent (%)**	**Cumulative percent (%)**
Caucasian/White	24	46.2	46.2
Hispanic	19	36.5	82.7
African American/Black	5	9.6	92.3
Other	4	7.7	100
Female	36	69.2	69.2
Male	16	30.8	100
Total	52	100	

### Post-measurement

There was a decrease in the total number of participants from (*n* = 52) to (*n* = 41) due to loss of follow-up for the post-measurement. Weight descriptive statistics were R = 113.5–374, average = 211.99, and SD = 65.29. The quartiles for weight were obtained as Q1 = 165.5, Q2 = 198, and Q3 = 246.75, respectively. The BMI variable had R = 19.82–56.59, average = 33.73, and SD = 9.44. The quartiles for BMI were obtained as Q1 = 27.23, Q2 = 32.28, and Q3 = 37.4.

The descriptive statistics for the LDL variable are as follows R = 53–221, average = 120.16, and SD = 34.43. The quartiles for LDL were obtained as Q1 = 99, Q2 = 113.5, and Q3 = 137. For HDL measurements R = 30–83, average = 54.4, and SD = 13.21. The quartiles for HDL were obtained as Q1 = 44, Q2 = 54, and Q3 = 65. For cholesterol there was R = 94–270, average = 176.58, and SD = 36.26. The quartiles for cholesterol were obtained as Q1 = 153.75, Q2 = 169.5, and Q3 = 201.

The descriptive statistics for the glucose variable are as follows R = 4.8–9.8, average = 5.65, and SD = 1. The quartiles for glucose were obtained as Q1 = 5.28, Q2 = 5.4, and Q3 = 5.63. The following are descriptive statistics (*n* = 41) for body fat percentage *R* = 19.4–57.3, average = 39.07, and SD = 10.84. The quartiles for body fat percentage were obtained as Q1 = 30.4, Q2 = 39.2, and Q3 = 47.2. For systolic blood pressure R = 96–174, average = 127.95, and SD = 17.09. The quartiles for systolic blood pressure were obtained as Q1 = 114.5, Q2 = 131, and Q3 = 139. For diastolic blood pressure R = 61–102, average = 83.39, and SD = 9.98. The quartiles for diastolic blood pressure were obtained as Q1 = 77, Q2 = 84, and Q3 = 92. The detailed descriptive statistics of the variables are given in Table 3.

**Table 3:** **Pre- and post- measurements of the participants—descriptive statistics**.

	**Number of subjects**	**Mean**	**Std. deviation**	**Percentile 25th**	**Percentile 50th**	**Percentile 75th**
Weight pre-test (LBS)	52	209.221	63.420	161.000	199.500	244.500
Weight post-test (LBS)	41	211.988	65.286	165.500	198.000	246.750
BMI pre	52	33.294	9.172	26.065	31.665	37.363
BMI post	41	33.729	9.445	27.230	32.280	37.395
LDL pre (mmol/L)	47	119.149	28.354	98.000	119.000	141.000
LDL post (mmol/L)	38	120.158	34.434	99.000	113.500	137.000
HDL pre (mmol/L)	47	50.170	12.291	40.000	51.000	61.000
HDL post (mmol/L)	38	54.395	13.206	44.000	54.000	65.000
Cholesterol pre (mmol/L)	47	178.702	33.281	162.000	175.000	201.000
Cholesterol post (mmol/L)	38	176.579	36.264	153.750	169.500	201.000
Glucose pre (mmol/L)	46	5.691	1.358	5.175	5.400	5.700
Glucose post (mmol/L)	38	5.653	0.9953	5.275	5.400	5.625
Body fat % pre	52	39.152	10.1269	30.825	40.700	47.150
Body fat % post	41	39.073	10.843	30.400	39.200	47.200
systolic blood pressure (bp) pre	52	126.538	16.453	113.000	132.000	136.000
Systolic BP post	41	127.951	17.089	114.500	131	139
Diastolic BP pre	52	81.308	10.011	73.250	82.000	88.500
Diastolic BP post	41	83.390	9.985	77.000	84.000	92.000

### Paired T-test

The Kolmogorov–Smirnov (K-Stats), test of normality was used to determine whether each variable for pre and post were normally distributed. We also checked the normality of each variable by Q-Q (Quantile-Quantile) plots and then used the paired samples *t*-test for each of the variables. By using the K-Stats and the Q-Q plots the following variables were determined as normally distributed: weight, LDL, HDL, cholesterol, percent body fat, and diastolic blood pressure. The glucose and systolic blood pressure variables did not meet the normality assumption even though statistical transformation was performed in order to use the paired samples *t*-test. When running the paired samples *t*-test, we used the subjects who completed both pre and post measurements.

The paired samples *t*-test was performed for the weight variable (*n* = 41) both for pre and post. There was a mean difference = 2.26 and standard error = 0.7 (95% CI: 0.85, 3.66). The test statistic *t*-value was equal to 3.245, *p* = 0.002. We conclude there was a significant difference between pre and post mean weights. The paired *t*-test for BMI followed, which was transformed for normality by a square root transformation due to some influential observations. There was a mean difference = 0.031 and standard error = 0.059 (95% CI: 0.01, 0.05). The test statistic *t*-value was equal to 3.37, *p* = 0.002. We conclude there was a significant difference between pre and post for BMI measurements.

Due to the inability to draw blood because of poor veins, loss to follow-up, or insufficient blood samples for analysis, the sample size decreased for the following variables: LDL (*n* = 36), HDL (*n* = 36), and glucose (*n* = 35). The *t*-test showed a mean difference for the LDL variable = −3.81 and standard error = 3.41 (95% CI: -10.73, 3.12). The test statistic *t*-value = −1.12, *p* = 0.272. There was no significant difference between pre and post mean LDL measurements. The *t*-test showed a mean difference for the HDL variable = −2.83 and standard error = 1.06 (95% CI: −4.98, −0.68). The test statistic *t*-value = 2.67, *p* = 0.011. We can conclude there was a significant difference between pre and post mean HDL measurements.

The mean difference for the cholesterol variable = 0.03 and standard error = 3.4 (95% CI: −6.87, 6.92). The *t*-test value was 0.008, *p* = 0.994; which indicates there was no significant difference between pre and post mean cholesterol measurements.

The percent body fat and the blood pressure measurements were obtained from all participants (*n* = 41) at the pre and post measurements. The paired *t*-test was performed for percent body fat (*n* = 41). There was a mean difference = 0.32 and standard error = 0.27 (95% CI: −0.22, 0.87). The test statistic *t*-value was equal to 1.2 with *p* = 0.236. There was no significant difference between pre and post mean percent body fat measurements. The paired *t*-test was performed for the diastolic blood pressure variable (*n* = 41). There was a mean difference = −2.39 with a standard error = 1.67 (95% CI: −5.76, 0.98). The test statistic *t*-value = −1.43, *p* = 0.160. There was no significant difference between pre and post-diastolic blood pressure measurements. The detailed paired samples *t*-test analysis for all participants is represented in Table 4.

**Table 4:** **Summary of statistical analysis in all participants - paired samples ***t***-test**.

	**Mean**	**Std. error mean**	**95% confidence lower**	**95% confidence upper**	***T*-value**	**Sig. (2-tailed)**
Weight (LBS)	2.256	0.695	0.851	3.661	3.245	0.002
BMI	0.359	0.107	0.142	0.576	3.344	0.002
LDL (mmol/L)	−3.806	3.411	−10.731	3.12	−1.116	0.272
HDL (mmol/L)	−2.833	1.06	−4.985	−0.682	−2.674	0.011
Cholesterol (mmol/L)	0.028	3.396	−6.866	6.922	0.008	0.994
Body fat %	0.324	0.27	−0.221	0.87	1.202	0.236
Diastolic BP	−2.390	1.67	−5.765	0.984	−1.432	0.160

### Females *T*-test

In this section, the following variables were found to be normally distributed LDL, HDL, cholesterol, and percent body fat. The paired samples *t*-test for weight (*n* = 28) was performed, which was transformed by the square root transformation due to some influential observations. The mean difference = 0.01 and standard error = 0.003 (95% CI: 0.003, 0.017). The test statistic *t*-value was equal to 2.883 with *p* = 0.008. There was a significant difference between mean weights among females. The paired *t*-test for BMI followed (*n* = 28), which was transformed by the square root transformation. The mean difference = 0.029 and standard error = 0.01 (95% CI: 0.009, 0.049). The test statistic *t*-value was 2.966, *p* = 0.006. We can conclude there was a significant difference between pre and post mean BMI measurements for females.

The paired samples *t*-test was performed for the LDL variable (*n* = 23). There was a mean difference = −2.83, standard error = 4.59 (95% CI: −12.33, 6.68). The test statistic *t*-value was −0.616, *p* = 0.544. There was no significant mean difference between pre and post LDL measurements among women.

The paired samples *t*-test was then performed for HDL (*n* = 23). The mean difference = −3.09 and standard error = 1.48 (95% CI: −6.15, −0.02). The test statistic *t*-value was equal to −2.09 with *p* = 0.049. We conclude there was a significant difference between pre and post mean HDL measurements for women. The paired *t*-test was performed for cholesterol (*n* = 23). The mean difference = 0.26 and standard error = 4.63 (95% CI: −9.34, 9.86). The test statistic *t*-value = 0.06, *p* = 0.956. There was no significant difference between pre and post mean cholesterol measurements among females.

The paired samples *t*-test was performed for percent body fat (*n* = 28). The mean difference = 0.09 and standard error = 0.30 (95% CI: −0.53, 0.71). The test statistic *t*-value was equal to 0.31 and *p* = 0.761. There was no significant difference between pre and post mean percent body fat measurements. The diastolic blood pressure variable (*n* = 28) was transformed by the square root of the variable. Mean difference = −0.12 and standard error = 0.11 (95% CI: −0.35, 0.10). The test statistic *t*-value was equal to −1.12, *p* = 0.273. There was no significant difference between mean diastolic blood pressure measurements. The detailed paired samples *t*-test analysis for females is represented in Table 5.

**Table 5:** **Summary of statistical analysis in female participants -paired samples ***t***-test females**.

	**Mean**	**Std. error mean**	**95% confidence lower**	**95% confidence upper**	***T*-value**	**Sig. (2-tailed)**
Weight (LBS)	0.01	0.003	0.003	0.017	2.883	0.008
BMI	0.029	0.01	0.009	0.049	2.966	0.006
LDL (mmol/L)	−2.82	4.585	−10.731	3.12	−1.116	0.272
HDL (mmol/L)	−3.087	1.478	−6.153	−0.021	−2.088	0.049
Cholesterol (mmol/L)	0.261	4.628	−9.337	9.859	0.056	0.956
Body fat %	0.093	0.302	−0.526	0.712	0.308	0.761
Diastolic BP	−0.122	0.109	−0.347	0.102	−1.119	0.273

### Males *T*-test

In this section, all the variables were found to be normally distributed. The paired *t*-test was performed for the weight variable (*n* = 13). The mean difference = 2.81 and standard error = 1.61 (95% CI: −0.69, 6.31). The test statistic *t*-value = 1.75, *p* = 0.106. There was no significant difference between pre and post mean weights among males. The paired samples *t*-test was performed for the BMI variable (*n* = 13). The mean difference = 0.39 and standard error = 0.23 (95% CI: −0.11, 0.90). The test statistic *t*-value was 1.71, *p* = 0.113. There was no significant difference between pre and post mean BMI measurements for males.

The paired samples *t*-test was performed for the LDL variable (*n* = 13). The mean difference = −5.54 and standard error = 5.04 (95% CI: −16.52, 5.44). The test statistic *t*-value was −1.1, *p* = 0.293.There was no significant difference between pre and post mean LDL. The paired samples *t*-test was performed for the HDL variable (*n* = 13). The mean difference = −2.38 and standard error = 1.4 (95% CI: −5.44, 0.66). The test statistic *t*-value = −1.71 with *p* = 0.114. There was no significant difference between pre and post mean HDL measurements.

The paired samples *t*-test was performed for the cholesterol variable (*n* = 13). The mean difference = −0.38 and standard error = 4.86 (95% CI: −10.98, 10.21). The test statistic *t*-value was equal to −0.08, *p* = 0.938. There was no significant difference between pre and post mean cholesterol measurements. The paired samples *t*-test was performed for the glucose variable (*n* = 12). The mean difference = 0.21 and standard error = 0.10 (95% CI: −0.02, 0.44). The test statistic *t*-value was equal to 1.99, *p* = 0.072.There was no significant difference between pre and post mean glucose measurements.

The paired samples *t*-test was performed for the percent body fat variable (*n* = 13). The mean difference = 0.82 and standard error = 0.54 (95% CI: −0.36, 2). The test statistic *t*-value = 1.52, *p* = 0.155. There was no significant difference between pre and post mean percent body fat measurements. The paired samples *t*-test was performed for the systolic blood pressure variable (*n* = 13). The mean difference = 0.62 and standard error = 4.34 (95% CI: −8.85, 10.08). The test statistic *t*-value was equal to 0.142, *p* = 0.890. There were no significant mean differences for systolic blood pressure. The paired samples *t*-test was performed for the diastolic blood pressure variable (*n* = 13). The mean difference = −2.85 and standard error = 3.26 (95% CI: −9.95, 4.26). The test statistic *t*-value = −0.873, *p* = 0.4. There was no significant difference between pre and post mean diastolic blood pressure measurements. The detailed paired samples *t*-test analysis for males is represented in Table 6.

**Table 6:** **Summary of statistical analysis in male participants - paired samples ***t***-test males**.

	**Mean**	**Std. error mean**	**95% confidence lower**	**95% confidence upper**	***T*-value**	**Sig. (2-tailed)**
Weight (LBS)	2.808	1.607	−0.693	6.309	1.747	0.106
BMI	0.394	0.230	−0.108	0.896	1.709	0.113
LDL (mmol/L)	−5.539	5.040	−16.520	5.444	−1.099	0.293
HDL (mmol/L)	−2.385	1.398	−5.432	0.662	−1.705	0.114
Cholesterol (mmol/L)	−0.385	4.863	−10.980	10.211	−0.079	0.938
Glucose (mmol/L)	0.208	0.105	−0.022	0.439	1.988	0.072
Body fat %	0.823	0.105	−0.022	0.439	1.988	0.072
Systolic BP	0.615	4.342	−8.845	10.076	0.142	0.890
Diastolic BP	−2.846	3.262	−9.953	4.261	−0.873	0.400

### Results of the survey

A behavioral survey, developed by Pace Projects^6^, was conducted during the initial biometric screenings. The survey was used to gain insight about the subjects' perception of exercise and nutrition as it relates to their health.

The survey responses indicate that 83% of the subjects agreed with the statement that they enjoyed physical activity. Yet when asked how they preferred to spend their leisure time, 56.6% preferred non-active activities, and 71.7% of the participants answered that many times, often, and sometimes they thought about how their surroundings affect the physical activity they do.

In addition, 24.5% indicated they never keep track of the physical activity they do, and ~21% of the study participants indicated that they never placed reminders around their home about physical activity.

The study participants viewed nutrition differently. They seemed to view physical activity and nutrition separately as a way to improve their overall health. They know the importance of eating fresh fruits and vegetables on their body, based on 86.7% participants' responses, and they intended, based on 79.2%, to eat at least 5 servings within 6 months; however, it seems that they are faced with the following barriers in regards to fresh fruits and vegetables: preparation (56.5% importance), cost (83% importance), and 76% are not satisfied by the fresh fruits and vegetables when eaten.

## Discussion

### GFL program

In 2011, Lubbock, Texas, was ranked the 3rd fattest city in the United States (Perron, 2011). Because of this ranking, the study intended to reduce chronic disease risk factors (cholesterol levels, glucose levels, and blood pressure levels) and decrease body weight, BMI, and body fat percentage by providing active living options through community walking clubs and healthy eating information through educational sessions.

Many risk factors contribute to the development of chronic diseases. Leading a sedentary lifestyle and being physically inactive can result in the development of over 35 chronic diseases (Booth et al., 2012). A primary form of prevention of these chronic diseases is exercise (CDC and U.S. Department Of Health and Human Services, Preventing Chronic Diseases, 2015). Merriam dictionary states that exercise is the physical activity that is done in order to become stronger and healthier. According to the World Health Organization (WHO)^7^, individuals between the ages of 18–64 should be involved in moderate intensity physical activity for at least 150 min per week, or individuals can exercise at a more vigorous intensity for 75 min per week in order to gain optimal health benefits (World Health Organization global recommendations of physical activity for health). Moderate physical activity includes walking, hiking, dancing, playing beginner level tennis, and gardening. Vigorous activity includes jogging, running, playing mid to high-level tennis, and push-ups. Research has also shown levels of physical activity and functional aerobic capacity each decline steadily with age (Flegal et al., 2005), while the prevalence of obesity tends to increase with age (Sui et al., 2007). It is also well documented that good nutrition contributes to both longevity and quality of life as we age. Recent studies have indicated that plant-based diets make the most significant changes in a person's overall wellness. Although a specific diet was not recommended during this study, portion control, salt in-take and the inclusion of fresh fruits and vegetables were highly recommended by a registered dietitian. While many know the harmful effects of obesity, finding motivation to combat weight gain is difficult for many individuals. Thus, health programs for adults should highly emphasize a combination of adequate active living and healthy eating options, in addition to developing successful ways to motivate individuals to adopt healthy behaviors and or lifestyle changes.

Since self-reporting physical activity by individuals has not always been reported accurately, (Troiano et al., 2007) this study incorporated a team-based approach that included a team captain that would verify their teammates' reported information. In addition, a sign in sheet was at each walking and educational session to verify attendance. In the study conducted by Manry, researchers did not conduct initial and final weigh-in sessions on all participants. In addition, that study did not ask participants if they had been diagnosed with diabetes or other co-morbid conditions. Furthermore, they did not obtain information related to the number of specific classes attended. Unlike that study conducted by Manry, this study captured biometric data from participants pre- and posts the intervention, asked if they had chronic conditions and asked all study participants to sign-in at each intervention (Manry and Peterson, 2013). Our results indicate that there was significant difference in weight, BMI, and HDL. Therefore, community based programs need to have some form of accountability to be effective (McLeroy et al., 2003).

Although several researchers have conducted similar studies that conclude the importance of physical activity and good nutrition, and the results from this study do indicate that the average exercise minutes per week was more than the recommended, study personnel observed a few subjects exercising less than the recommended amount. Study personnel would encourage the subjects to exercise for at least 30 min at a brisk pace. In addition, at one of the education sessions, participants inquired about fat free vs. reduced fat. The dietitian recommended the importance of portion control while eating either fat free or reduced fat. The participant was unhappy with the dietitian's recommendation to begin with smaller portions. While participants were receiving information from highly reputable sources, (the WHO for recommended exercise time and a registered dietitian), the participants did not seem willing to make adjustments to their physical activity and/or their dietary needs. By not adjusting the recommendations based on reputable sources, individuals faced with modifiable risk factors may develop deadly diseases that are preventable. In previous years, the WHO stated that in order to combat physical inactivity related to disease, a social ecological approach is needed to combat physical inactivity as it relates to the global burden of disease, death, and disability. However, more recent research suggests that combating physical inactivity requires a change in delivery through environmental and policy adjustments. As a result the social ecological model must be upended into a structure that focuses more on policy change relevant to its environment, establishing sustained local collaborations and networks, and providing equitable services (Golden et al., 2015).

Strengths of this study included pre and post study design and the number of teams (*n* = 4) that were used to keep the participants motivated. The limitations of the study include the small sample size, lack of control group in the study design, and lack of nutrition tracking that could have produced some biases.

Our study findings may have implications to the education, exercise and socio-economic status in Lubbock residents. Our research has concluded that individuals in low-income neighborhoods and disparate communities have environmental barriers that prevent them from active living and healthy eating (Brownson et al., 2001). Therefore, based on how the subjects from this study answered the questions, participants might prefer to spend their leisure time on physical activity if their surroundings were improved since they do enjoy physical activity.

In addition, because several people do not feel it's important to track and put reminders and they don't look for information about exercise, many subjects may have been in pre-contemplation stages and are not forming habits. Habit formation is crucial for behavior change (Stawarz et al., 2015).

## Conclusion

Based on these findings, we conclude that the intervention showed positive effects on exercise and lifestyle. The data among males was found as normal while females had some variables with influential observations, which were transformed for analysis. For future studies, we will include an increase in study participants, inclusion of cognitive assessment(s), and an environmental component. Environmental barriers do play a key role in staying active and eating healthy foods. However, people will not change behavior if their environment does not provide an opportunity for physical activity and accessibility to healthy foods.

## Author contributions

AB conception, design, and implementation of the study; submission of protocol to the IRB; drafting/revising/final approval of the manuscript. HK data analysis and interpretation of data. TL implementation of the study, acquisition of data and interpretation of data; assisted with the submission of protocol to the IRB; revision of manuscript. VM design, and implementation of the study; assisted with the submission of protocol to the IRB; revision of manuscript. JA design, and implementation of the study; assisted with the submission of protocol to the IRB; revision of manuscript. KT implementation of the study and revision of the manuscript. KS implementation of the study and revision of the manuscript. RG implementation of the study and revision of the manuscript. PR submission of protocol to the IRB; drafting/revising/final approval of the manuscript.

### Conflict of interest statement

The authors declare that the research was conducted in the absence of any commercial or financial relationships that could be construed as a potential conflict of interest.

## Footnotes

^1^*The State of Obesity. Better Policies for a Healthier America. A project of the Trust for America's Health and the Robert Wood Johnson Foundation*. Available online at: www.stateofobesity.org (Accessed September 30, 2015).

^2^*American Diabetes Association. In My Community*. Lubbock, Texas. Available online at: http://www.diabetes.org/in-my-community/local-offices/lubbock-texas/ (Accessed November 15, 2015).

^3^*Centers for Disease Control and Prevention Division of Nutrition, Physical Activity and Obesity. Adult Obesity and Consequences*. Available online at: http://www.cdc.gov/obesity/adult/causes.html (Accessed September 30, 2015).

^4^*American College of Sports Medicine*. Available online at: https://www.acsm.org/about-acsm/media-room/acsm-in-the-news/2011/08/01/acsm-aha-support-federal-physical-activity-guidelines (Accessed November 15, 2015).

^5^*U.S. Census Bureau. QuickFacts United States*. Available online at: http://quickfacts.census.gov/qfd/states/48/4845000.html (Accessed November 1, 2015).

^6^*Pace Projects*. Available online at: www.paceproject.org (Accessed June 1, 2015).

^7^*WHO Organization Global Recommendations of Physical Activity for Health*. Available onlineat: http://www.who.int/dietphysicalactivity/factsheet_recommendations/en/ (Accessed November 1, 2015).

## Appendix

Questionnaire:

The information sheet included the following questions:

1. Have you had a ≥10% change in weight in the past 3 months?
2. Have you had any types of weight loss surgery?
3. Are you able to perform exercise?
4. Are you participating in another weight loss program?
5. Are you taking or planning on taking any nutritional supplements that may affect muscle mass?
6. Are you taking or planning on taking any weight loss pills?
7. Do you currently have any chronic diseases?

In order to qualify for the research study, individuals had to fit the following criteria: (a) be able to participate in the GFL program for 8 weeks, (b) understand the purpose of the study, fill out the participation information form and sign a consent document, and (c) be between the ages of 18-89. Individuals were excluded from research if any of the following occurred:

1. individuals were unable or unwilling to provide a blood sample,
2. individuals had at least a 10% change in weight in the 3 months prior to the program,
3. individuals had any type of weight loss surgery, and
4. individuals diagnosed with the following chronic diseases: including but not limited to, cardiovascular disease, heart failure, blood clotting, asthma, and epilepsy.
